# Experiences of Caregivers in De-addiction Centers in Western Maharashtra, India: A Qualitative Study

**DOI:** 10.7759/cureus.68155

**Published:** 2024-08-29

**Authors:** Ram Prakash B U, Kajal Shrivastava, Dr. Hetal Rathod, Rohon Saha, Sweety Kumari

**Affiliations:** 1 Department of Community Medicine, Dr. D.Y. Patil Medical College, Hospital and Research Centre, Dr. D.Y. Patil Vidyapeeth, Pune, IND

**Keywords:** occupational health, satisfaction, burden, challenges, caregivers

## Abstract

Introduction

Engaging in caregiving can be incredibly gratifying for caregivers, but it can also lead to unfavorable outcomes and impose physical and psychological burdens on them. With limited research on the experiences and difficulties encountered by caregivers in de-addiction centers, this study primarily aims to explore de-addiction centric caregiver’s experiences and challenges.

Methodology

A qualitative study design with a phenomenological research method was adopted. An in-depth semi-structured interview was conducted from June to August 2023 in Hindi and English at the de-addiction centers where caregivers were employed. Interviews were audio recorded for further analysis.

Results

The interview results were thematically analyzed. Out of 12 Caregivers interviewed, eight were males and four were females, each representing various professions such as administrators, nursing staff, counsellors and helpers. Some of the emerging themes in our study are caregivers' perspective, the Burden among caregivers, and the emotional aspect. The findings indicated that five caregivers had previously treated alcohol dependents and family members of alcohol dependents, with a major part of caregivers expressing job satisfaction and finding fulfillment in helping patients overcome addiction, which brought them a sense of fulfillment in their lives. Other findings include two caregivers who faced financial challenges and often received insufficient salaries due to inadequate funding. Extended working hours, driven by patient needs, resulted in sleep deprivation, potentially causing physical health issues and emotional exhaustion among caregivers. Also, caregivers were frequently held in high esteem by the family members of patients and the family members of caregivers for providing unwavering support and encouragement.

Conclusion

Acknowledging the vital contributions of caregivers is pivotal for their well-being. It is a shared responsibility within the community to provide caregivers with emotional support, recognition, appreciation, and employer support. These gestures can make a substantial difference in the lives of caregivers and enhance the quality of care they offer to patients.

## Introduction

The healthcare industry has seen a significant change in the last several decades, characterized by a move away from institutionalization and toward the adoption of community-based initiatives and shorter hospital stays. In addressing drug misuse disorders, there is a heightened social awareness that permeates the individual, interpersonal, and societal layers of the care landscape. This change is especially visible in this regard [1,2]. Most importantly, this paradigm change emphasizes how important caregivers are as essential players in the healthcare system. The current range of reported caregiver burden, which is between 23.0% and 59.2%, emphasizes how important it is to comprehend and manage the difficulties experienced by caregivers [3,4]. Although providing care may be an extremely fulfilling endeavor, it is important to understand that it can also result in negative consequences, imposing both physical and psychological burdens on caregivers.

What sets this study apart is its unique focus on the challenges and experiences faced specifically by caregivers in deaddiction centers. Despite the global impact of substance abuse, there exists limited research on the specific challenges faced by caregivers in this context. By delving into the deaddiction-centric caregiver's experiences, this study aims to provide comprehensive insights beyond the existing knowledge base, contributing to a more subtle understanding of the complexities involved in caregiving within deaddiction settings. Ultimately, the research seeks to inform targeted interventions and support mechanisms tailored to caregivers' distinct needs in this critical healthcare.

## Materials and methods

A qualitative study design with a phenomenological research method was adopted. In-depth interviews were conducted from June to August 2023. at the premises of the deaddiction centers where caregivers were employed. The target population respondents are the caregivers working in deaddiction centers. Informed written consent was taken from the caregivers. The respondents who participated in the research were voluntary. The research participants were given an overview of the purpose of the study and assured that the information gathered through them will be confidential and used only for research purposes. After acquiring the participants' written informed consent, the respondents' basic information was documented, and the interview was audio recorded. Despite participants declining audio recordings, the investigators carefully documented the discussions by taking comprehensive notes. Institute of Ethical Committee Clearance (IESC/PGS/2023/198) was obtained before the start of the study from the Institutional Review Board in Dr. D.Y. Patil Medical College and Hospital, Pune. Data analysis was done using NVIVO 14 software [5].

## Results

The total interview duration recorded was 244 minutes, with an average time of 21 minutes per interview. Out of 12 caregivers interviewed, eight were males, and four were females, each representing various work, such as administrators, nursing staff, counsellors, and helpers. Among the caregiver's marital status, seven were single and five were married.

Out of the 12 participants interviewed, five individuals who worked in a deaddiction centre as caregivers successfully recovered from alcohol addiction and all five shared common characteristics such as their marital status, relationship with family members, previous job employment, and job satisfaction. None of the caregivers were married and mentioned that...“My family said you will get married once you get out of this addiction…time went by I couldn’t get out of this habit and it didn’t happen”.

With the previous job, all had problems with the employees due to alcoholism, and eventually, they all stopped working at one point in time. As caregivers are recovered addicts, they said that they found their work fulfilling and self-satisfying as they helped patients overcome addiction. All caregivers had trust issues with their family members when they were addicts, but now, they feel that their family members are proud of the service they are doing now.

**Figure 1:**
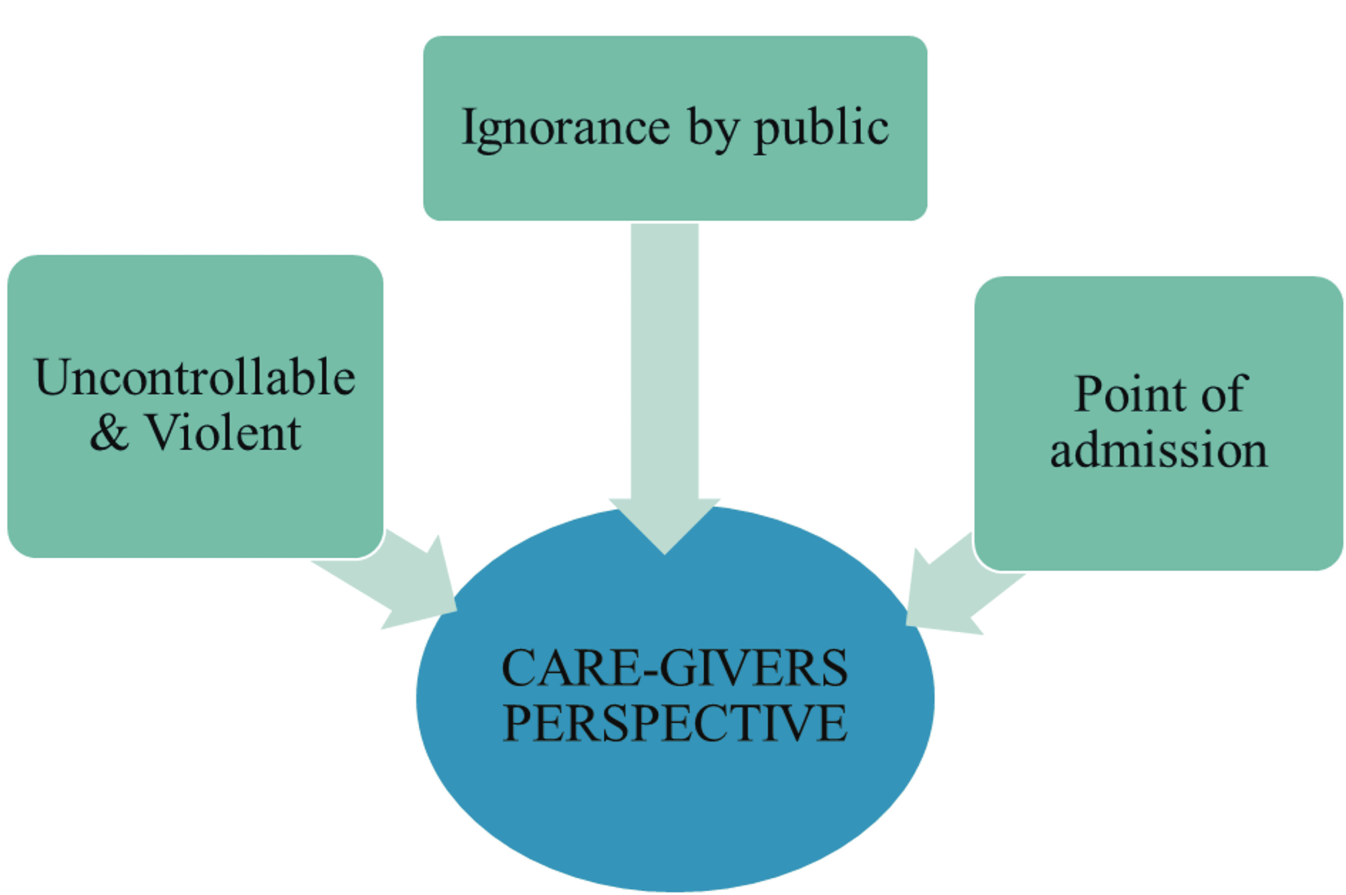
Caregiver's perspective Image credit: Ram Prakash

In our study, we identified three emerging themes: Caregiver's Perspective, Burden Among Caregivers, and the Emotional Aspect. From the Caregiver's perspective, many reported dealing with uncontrollable and violent behaviors, compounded by a sense of ignorance from the public. The point of admission, as illustrated in Figure 1.

Caregiver's perspective

Ignorance by the Public

A nurse aged 35 years was asked about what the public thinks of the job, the answer was-

“Till now society doesn’t consider addiction as a disease and patients to be human beings”.

another helper when asked about the same-

“Even sometimes they don’t consider me to be a nurse and telling why are you still working there?”

Uncontrollable and Violent

A Caregiver who works as a nurse aged 48 years when asked about how patients behave told-

"As the treatment progresses patients will become uncontrollable and violent and as a female nurse sometimes I can’t single-handedly manage the situation….I need help”.

Point of Admission

When asked about when patients come at the time of admission, a 65-year-old manager told-

“It's the most vulnerable point when a patient visits us for the first time, they will be in the worst phase of their life and more than that for their family members it is like the last hope for them”.

The same was told by another manager aged 58 years-

“They are helpless…sometimes they think they don’t need treatment they think they are perfectly ok but you can see it in their face”.

Burden among caregivers

**Figure 2:**
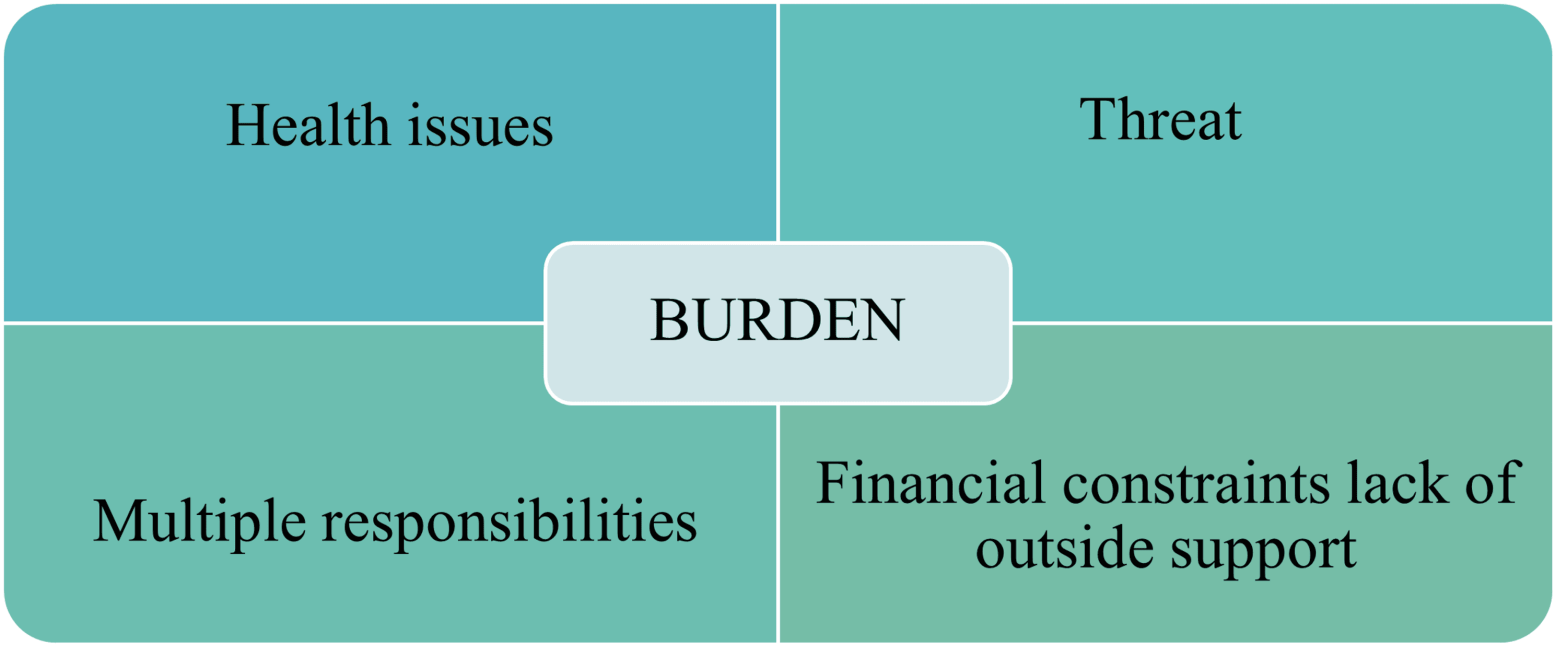
Burden among caregivers Image credit: Ram Prakash

In the theme Burden Among Caregivers, we identified subthemes as Health issues, Threat, Multiple responsibilities and Financial constraints lack of outside support (Figure 2).

Health Issues

A 35-year-old female nurse when asked about their sleep cycle told

“So most of the nights we are unable to sleep because every other day some patients will be having panic attacks, some will be screaming,….. So, we have to rush to stabilize the patient doing all this most of the night will be gone”.

A helper who is 34 years old said the same,

“Whenever we will be sitting to eat may it be lunch or dinner a sudden scream will come and everyone will be rushing leaving the food there itself so 2-3 hours are gone easily and our meals skipped and this way we lose our appetite”.

Multiple Responsibilities

Caregiver who has been working as a helper told,

“We are doing multiple shifts and this centre is easily accessible for poor people to get treatment, the caregivers here are much less compared to patients, so each will have multiple responsibilities”.

Threatening

A 24-year-old female counsellor replied when asked about the workplace environment-

“Most of the time patients will be nice they listen to what we tell, and share their thoughts & feelings with me but sometimes things are not as it is they will raise their voices “How can a little girl like you teach me life?”

Financial Constraints and Lack of Outside Support

When asked about the economic support, the manager of the centre replied to us-

“Being an NGO it's becoming hard for us day by day to maintain the expenses so we want the public to be more aware about this corner of society where contributing a little will bring a difference in patient care”

Another administrator aged 54 years replied the same,

“………for us, the money we are getting is so less that it becomes even more difficult for us to give money to the people working here that they deserve”

Emotional aspect

**Figure 3:**
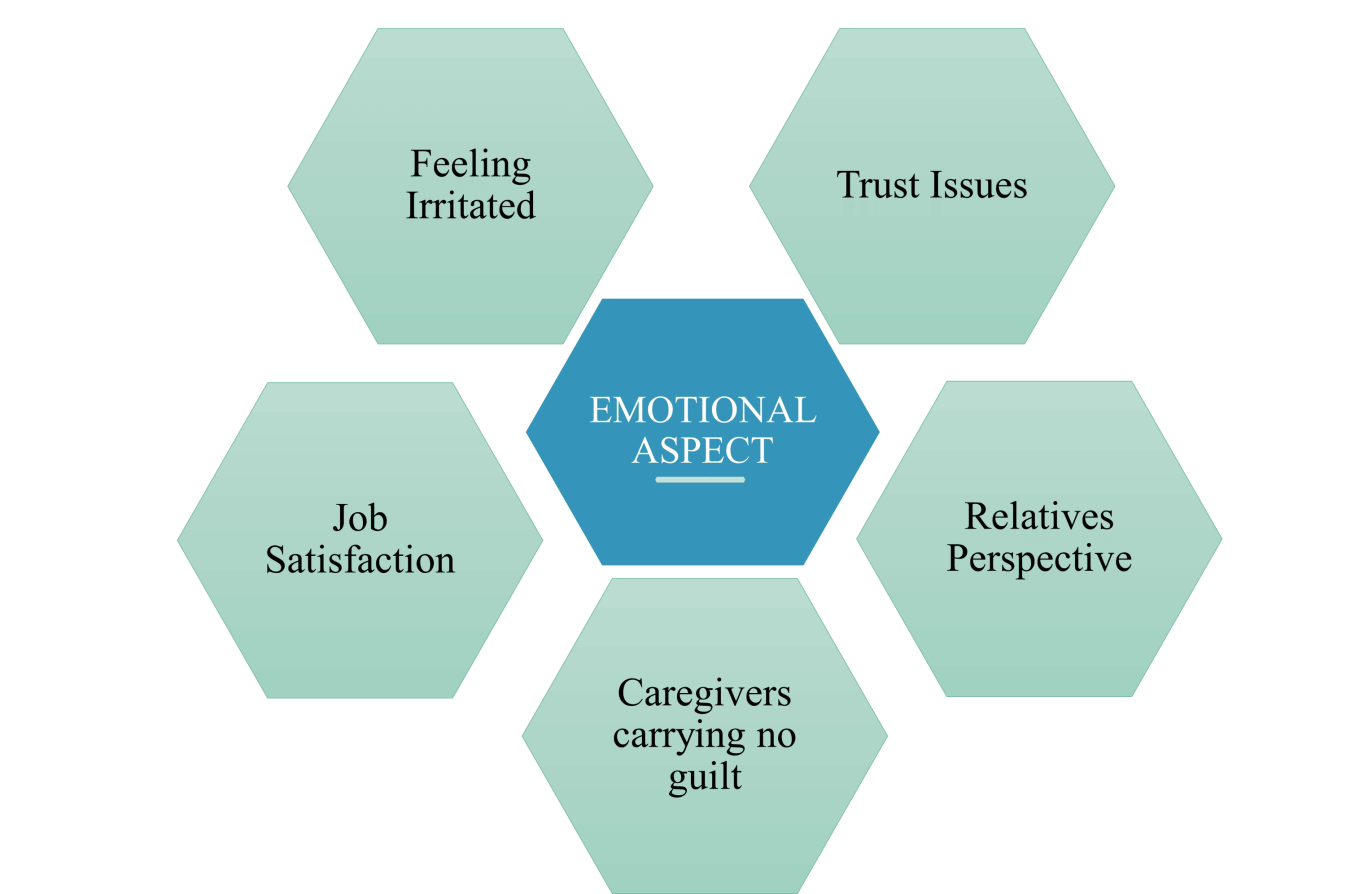
Emotional aspect among caregivers Image credit: Ram Prakash

In the theme Emotional Aspect, we identified subthemes as Feeling Irritated, Trust issues, Relatives Perspective, Job satisfaction and Caregivers carrying no guilt (Figure 3)

Caregivers Carrying no Guilt

As most of the caregivers will have caregivers guilt but the centres were we did the study are all saying it dosen’t help with treatment and they should always be strict with patients. One of the caregivers who work as a helper aged 41 years told-

“ Many of the times patients will be like “sir, please for the sake of god one last time arrange atleast one cigaratee, one quarter of alcohol” some will be like telling emotional stuff like “I lost my mother on this day atleast for this day arrange me something”.

Job Satisfaction

Since most of the caregivers are previous addicts, they told that they get a fulfilment in job by helping the patients. One of the managers working aged 58 years told

“Sir….someone helped me when I was exactly in this situation (crying) ………so I want to extend my hand to those victim because I know through what pain they are going right now”.

and also, a caregiver working as administrator aged 64 years told the same,

“It's not a job for me.…it's like something inside me which will not let me leave them”.

Trust Issues

All the Caregivers in the centre told they should trust the process of treatment but gaining the trust of patient is difficult and a 38-year-old doctor feels-

“Sometimes we develop so much of bonding with some patients and the very next day they are like they don’t even know us we are like strangers to them…. they will shout at us…..they will refuse to take medicines they will throw things at us, but doctors we definitely know through what phase they are going so its very difficult to gain their trust”.

Family Viewpoint

When we asked Caregivers about how the patient's family members treat them, they became emotional before they replied. A helper aged 34 years replied-

“To the patient's relatives, we are something next to God, whenever they come, they will burst into tears.… they believe that more than medication we are responsible for the recovery of their son/father/brother”.

Another caregiver working as a manager told-

“There was one of our patient's mom, who used to specifically go to mandir (temple) every Monday and she used to give pooja (prayer) for all of us along with her son and used to bring prasad (blessed food from the temple) for us and our family till her son's recovery”.

## Discussion

According to the findings of the study, a significant number of caregivers were ex-addicts. Caregivers also face financial difficulties, mental and physical problems, and other challenges, but they also report feeling satisfied with their jobs when assisting patients. Similar findings were discovered in research done by Saha et al. in 2023 on caregiver burden, which demonstrated that a lack of social activities, conflicting responsibilities, and inadequate financial resources are the antecedents of caregiver strain [2]. Financial and economic constraints have a significant role in the stress of caregiving. Even if the government offers financial support to patients with chronic diseases to lessen the caregivers' load, caregivers facing financial and economic strain could nonetheless bear an increased amount of responsibility [6]. A study done by Kaur et al. in 2018 mentioned that caregiver is burdened more when their profession, caregiving obligations, and family needs conflict [7]. After assigning a score based on the scale, the respondents' overall burden level was as follows: 68% of the respondents reported moderate to severe burden, 18% reported severe burden, and 14% reported mild to moderate burden [7].A study done by Liu et al. in 2020 that examined the benefits of caregiving for patients with opioid dependence and its relationship to quality of life, social support, and caregivers' burden suggested that caregivers may also benefit from some aspects of caregiving. Among Scale for Positive Aspects of Caregiving Experience (SPACE's) four categories, motivation for the job of caregiver received the highest rating, followed by caregiver satisfaction, caregiver personal benefit, and social elements of caregiving [8].

Acknowledging the critical roles that caregivers play is vital not only for their own well-being but also for the overall success of care they provide. When caregivers feel valued and supported, they are better equipped to manage the emotional and physical demands of their responsibilities. This recognition can lead to improved mental health, reduced burnout, and enhanced quality of care, ultimately benefiting both the caregivers and those they care for [9]. It emphasizes the difficulties faced by caregivers on a personal, emotional, psychological, and social level in addition to the activities involved in providing care. A study done by Rajpurohit et al. in 2023, caregivers have challenges in their daily lives in addition to the caregiving load [9]. One important aspect brought up is that caregivers frequently don't have enough emotional outlets or ventilation when providing care. This shows that caregivers may feel stressed out and emotionally drained because their job is so demanding. Furthermore, the study mentions that particularly when working with patients who exhibit addictive tendencies, caregivers may encounter unfavorable views, embarrassment, and even hostility from others [10]. These issues must be acknowledged and resolved for caregivers' overall well-being. It promotes a shared obligation among the community to offer emotional support, acknowledgment, gratitude, and employer assistance to caregivers. These actions are thought to be crucial to increasing the quality of care that caregivers offer patients and having a significant impact on their lives [11]. Additionally, emphasizes the value of social workers in helping people-including caregivers-who may otherwise be shunned by society. Social workers are defined as devoted professionals whose mission is to advance people's well-being in a variety of life circumstances. Most caregivers suffer moderate to severe burden that can significantly affect them and the care for the patient [12].

Study limitations

The caregivers in the study come from various professions, making it challenging to apply a common burden across all participants. Due to the small sample size, the results cannot be generalized. Additionally, caregivers were selected based on their availability at the center during data collection, leading to an unequal representation of different professions in the sample.

## Conclusions

In conclusion, the study underscores the urgent need for a comprehensive understanding of caregiver burden, which extends far beyond the logistical demands of caregiving. The emotional, psychological, and social challenges that caregivers grapple with daily often go unnoticed. Yet, they have profound implications on their well-being and, consequently, on the quality of care they provide. Caregivers endure significant stress, emotional exhaustion, and social isolation, all of which can lead to adverse health outcomes if left unaddressed. It is therefore crucial that healthcare professionals and society at large recognize the multifaceted nature of caregiver burden. Addressing this burden necessitates a holistic approach that includes access to mental health resources, respite care options, support groups, and comprehensive training for caregivers. By implementing these support mechanisms, we not only empower caregivers to perform their roles more effectively but also safeguard their health and well-being. This study's significance lies in its call to action: to create a more supportive environment for caregivers, one that acknowledges their indispensable contributions and alleviates the pressures they face. A society that invests in the well-being of its caregivers not only improves the lives of those who dedicate themselves to the care of others but also enhances the overall quality of care within the healthcare system. Ultimately, the well-being of caregivers is inextricably linked to the well-being of those they care for, making this issue one of paramount importance.
